# Editorial: Molecular biomarkers of cardiometabolic disease - volume II

**DOI:** 10.3389/fendo.2026.1792935

**Published:** 2026-01-30

**Authors:** Mirjana Macvanin, Aleksandra Klisic

**Affiliations:** 1 Institute of Chemistry, Technology and Metallurgy, Center for Chemistry, National Institute of the Republic of Serbia, University of Belgrade, Belgrade, Serbia; 2 Department for Biochemistry, University of Montenegro-Faculty of Medicine, Podgorica, Montenegro; 3 Center for Laboratory Diagnostics, Primary Health Care Center, Podgorica, Montenegro

**Keywords:** cardiometabolic disease, cardiovascular disease, diagnostic marker, insulin resistance, molecular biomarker, prognostic biomarker, risk assessment, type 2 diabetes

Cardiometabolic disease (CMD), a pathophysiological process that leads to the concomitant occurrence of cardiovascular and metabolic diseases, remains a principal global cause of mortality and morbidity. Progress in CMD research remains hindered by the limited predictive capabilities of classical risk factors, necessitating innovative approaches to identify molecular biomarkers that enhance CMD screening, diagnosis, and prognosis. This Research Topic extends the efforts outlined in our first Research Topic, published in Frontiers of Endocrinology in 2024 (Macvanin et al.), where we initiated the assembly of innovative original research and review papers on the identification of novel CMD biomarkers and their evaluation for prognostic and diagnostic use.

Of particular interest were papers that used innovative approaches, including high-throughput Omics methodologies and machine learning, to investigate the genetic and biochemical bases of CMD-associated complications, including insulin resistance (IR), type 2 diabetes (T2DM), and cardiovascular disease (CVD).

IR, which includes defective glucoregulation and inflammation accompanied by perturbed secretion of adipose tissue factors and endothelial dysfunction, is central to CMD pathogenesis. Peng et al. investigated the effect of glycemic variability (GV) on patients with mitral valve disease. The data indicate that elevated GV may reliably predict short-term mortality, suggesting that timely monitoring and management of GV can improve clinical outcomes in this high-risk patient group.

The prognostic value of the Stress Hyperglycemia Ratio (SHR) is investigated in a large cohort of patients with coronary disease in a study by Yan et al. The authors studied the relationship between SHR and 1-year outcomes after Acute Myocardial Infarction (AMI) and showed a significant positive association between SHR and 1-year clinical outcomes in patients with AMI.

A retrospective cohort study of elderly HF patients investigated the correlation between neutrophil gelatinase-associated lipocalin (NGAL), the monocyte-to-high-density lipoprotein cholesterol ratio (MHR), and angiotensin II (Ang II) with short- and long-term mortality. Liu et al. report that MHR may represent a robust and reliable predictor of mortality in the elderly HF patients.

A study by Lian et al. investigated the cumulative effects of two biomarkers implicated in the development of CVD: atherogenic index of plasma (AIP), a biomarker of lipid dysregulation, and high-sensitivity C-reactive protein (hs-CRP), a well-known marker of inflammation. The authors reported an association between increased AIP and hs-CRP levels and CVD, reflecting an interplay between lipid metabolism and inflammation in CVD pathogenesis.

A study by Jian et al. investigated the prognostic effects of an atherogenic lipid component, remnant cholesterol (RC), in patients with acute decompensated heart failure (ADHF). RC is implicated in the pathogenesis of cardio- and cerebrovascular diseases, and a cohort study by Jian et al. demonstrates a dose-dependent relationship between RC and 30-day mortality in ADHF patients.

A study by Liu et al. employed differential expression analysis and machine learning to identify potential diagnostic biomarkers and therapeutic candidates for metabolic syndrome-associated myocardial ischemia-reperfusion injury (MetS-MIRI). The authors found that immune-metabolic dysregulation may serve as a key driver of MetS-MIRI and propose several biomarkers for its diagnosis.

Sestrin2, a stress-inducible antioxidant protein, may play a complex and context-dependent role in metabolic regulation. A cross-sectional study by Zahid et al. investigated the relationship between plasma Sestrin2 levels and various cardiometabolic indices in both healthy and diabetic subjects and found that Sestrin2 may serve as a protective factor in healthy individuals but may exacerbate metabolic perturbations in diabetes.

Wang et al. investigated potential associations between indicators of liver function and atherosclerotic cardiovascular disease (ASCVD) and reported that hepatocyte damage markers, including aspartate aminotransferase, alanine aminotransferase, gamma-glutamyl transferase, alkaline phosphatase, and total bilirubin, were risk factors for ASCVD, whereas albumin showed a protective effect.

A study by Sun et al. reports a positive correlation between the Fibrosis-4 Index (FIB-4) and myocardial infarction (MI) in Chinese patients with T2DM and proposes that the FIB-4 index may serve as a promising tool for early MI detection in hypertensive individuals.

Research paper by Lin et al. identified potential biomarkers for predicting amlodipine therapeutic efficacy in pediatric primary hypertension (PH), which represents a dominant type of hypertension seen in children and adolescents. The study reported that the therapeutic efficacy of amlodipine was negatively associated with hyperinsulinemia, IR, and amlodipine concentrations, whereas a positive association was observed with plasma endothelin-1 (ET-1).

Cybulska et al. identify a peptide hormone, adiponectin, as a promising biomarker for predicting cardiometabolic risk in postmenopausal women. A cross-sectional study of perimenopausal women showed that adiponectin correlated positively with HDL-c and negatively with glycated hemoglobin, fasting blood glucose, insulin, and triglycerides. Thus, the findings of this study suggest an association between adiponectin and specific markers of metabolic health, such as IR, visceral fat distribution, and lipid profile.

Yang et al. proposed that the inflammation biomarker neutrophil-to-lymphocyte ratio (NLR) may serve as a potential indicator for identifying left ventricular diastolic dysfunction (LVDD) in T2DM patients. A cross-sectional study of a large number of T2DM patients reported that T2DM patients with elevated NLR levels may be at a greater risk of developing LVDD than those with lower NLR levels.

Huang et al. investigated the relationship between the AIP and serum uric acid (SUA) levels. The authors find that AIP correlates with hyperuricemia risk and is significantly associated with SUA levels in a non-linear fashion. These findings may improve clinical approaches to managing metabolic and cardiovascular risks associated with increased AIP.

Our Research Topic also encompasses four reviews. A review article by Chen et al.analyzed cardiac energy metabolism (e.g., mitochondrial oxidative phosphorylation and regulation of glucose and lipid metabolism) and their dysregulation in disease states, while evaluating intervention strategies targeting metabolic pathways, such as mitochondrial function enhancement and modulation of substrate utilization. A review by Liu et al. discusses the roles of ferroptosis and mitochondrial damage in the onset and progression of diabetic cardiomyopathy (DCM), whereas a systematic review by Abdelhameed et al. analyzes associations between circulating angiopoietin-like protein 8 (ANGPTL8) concentrations and steatotic liver disease associated with metabolic dysfunction. A review by Liu et al. provides a timely update on the research progress investigating the relationship between the free fatty acids profile in T2DM and the occurrence of vascular complications.

We hope that the papers presented in this Research Topic will inspire further exploration of novel research and clinical trends in the identification, characterization, and assessment of molecular biomarkers of CMD. We are excited to witness future efforts to implement a refined systematic assessment of potential novel CMD biomarkers for clinical application.

